# *Vegfr3* and *Tbx1* Interact in Cardiac Morphogenesis

**DOI:** 10.3390/jcdd13080399

**Published:** 2026-08-20

**Authors:** Stefania Martucciello, Marchesa Bilio, Sara Cioffi, Ilaria Aurigemma, Mariangela Cavallaro, Antonio Baldini, Elizabeth Illingworth

**Affiliations:** 1 Department of Chemistry and Biology “Adolfo Zambelli”, University of Salerno, 84084 Fisciano, Italy; iaurigemma@unisa.it (I.A.); cavallaromariangela2@gmail.com (M.C.); 2 Institute of Genetics and Biophysics “Adriano Buzzati-Traverso”, National Research Council, 80131 Naples, Italy; marchesa.bilio@igb.cnr.it (M.B.); sara.cioffi@igb.cnr.it (S.C.); bldntn@gmail.com (A.B.)

**Keywords:** outflow tract, genetic interaction, congenital heart disease, mouse model, *Tbx1*, *Vegfr3*

## Abstract

Gene inactivation in model organisms has identified numerous genes and signaling pathways involved in mammalian cardiac outflow tract (OFT) development. Human genetics data have implicated the *VEGFR3* gene in OFT development, but when and where it is required is unknown. In this study we determined the sensitivity of the developing murine heart to reduced *Vegfr3* gene dosage, and we tested whether its requirement is dependent upon TBX1, a known regulator of *Vegfr3* expression in cardiac and lymphatic endothelial cells. We found that in the mouse, a single copy of the *Vegfr3* gene was sufficient for normal heart development in most cases. Mutation of a single copy of the *Tbx1* gene greatly enhanced the sensitivity of heart development to *Vegfr3* dosage reduction and led to the formation of cardiac defects. In addition, deletion of *Vegfr3* in the *Tbx1* expression domain also led to severe cardiac OFT abnormalities. We used RNAscope to reveal the location of *Vegfr3* and *Tbx1* transcripts in midterm mouse embryos. This revealed co-localization of these transcripts in the endothelium of the aortic sac, caudal pharyngeal arch arteries and proximal OFT, suggesting that these are potential sites of genetic interaction between *Vegfr3* and *Tbx1* that are critical for murine heart development.

## 1. Introduction

Mutations of the *VEGFR3* and *TBX1* genes are associated with Milroy syndrome and DiGeorge syndrome (DGS), respectively. Both diseases affect the vascular system. Milroy syndrome is the most frequent primary hereditary lymphedema [1]. Studies in animal models have shown that *Vegfr3* is a major lymphangiogenesis gene, being required for the formation of the jugular lymph sacs and systemic lymphatic vessels [2,3,4]. Prior to the development of the lymphatic system, *Vegfr3* is required in blood vessels, and its loss causes early embryonic lethality due to the failure of remodeling of the primitive vascular plexus, which requires angiogenic sprouting [5,6]. DiGeorge syndrome, on the other hand, is primarily a *TBX1* haploinsufficiency disorder, and is not commonly associated with lymphatic anomalies, although rare cases have been reported [7,8]. In humans, DGS is mainly caused by a heterozygous chromosomal microdeletion on chromosome 22 that encompasses over 40 genes, including *TBX1*. Heterozygous *TBX1* point mutations recapitulate most of the physical defects associated with the 22q11.2 chromosomal microdeletion, including congenital heart defects, skeletal defects, facial dysmorphism and immune defects, thus providing evidence of the pleotropic functions of TBX1 during embryonic and fetal development [9].

Studies in mice have shown that TBX1 plays a critical role in cardio-pharyngeal mesoderm (CPM), a population of progenitors that differentiates into different cell types that contribute to the formation of the heart, facies and neck glands [10], thereby accounting for the aforementioned clinical signs and symptoms of DGS. Furthermore, homozygous inactivation of *Tbx1* causes generalized lymphatic vessel hypoplasia or aplasia, which is lethal perinatally [11]. TBX1 activates *Vegfr3* expression in murine lymphatic endothelial cells by binding a putative enhancer in the endogenous *Vegfr3* gene [11], and the two molecules interact strongly during cardiac lymphangiogenesis [12]. TBX1 also regulates *Vegfr3* in brain microvessels during murine embryogenesis [13].

In humans, rare variants of the *VEGFR3* gene are associated with cardiac outflow tract (OFT) abnormalities, including Tetralogy of Fallot (TOF) [14,15,16,17], suggesting a previously unknown role for the gene in cardiac development. Interestingly, TOF is the most common congenital heart defect found in patients with DGS [18,19], and at least some of the hallmarks of TOF occur in mouse models of DGS [20]. We hypothesize that interaction between TBX1 and *Vegfr3* is required in cardiac progenitors in the CPM, or in their descendants, for development of the cardiac OFT. Studies conducted in mice, including mouse models of DGS, show that TBX1 is required for the deployment of cardiac progenitors from the second heart field (part of the CPM) into the developing OFT [21,22]. This progenitor population gives rise to cardiomyocytes, endothelial cells (EC) and branchiomeric myocytes [23,24,25]. Fate mapping experiments have revealed that ECs in the aortic sac (AS) and OFT are of second heart field (SHF) origin [26,27], but they have also revealed the existence of different populations of SHF progenitors that are spatially and temporally separate and give rise to different structures and cell types. For example, ECs of the anterior pharyngeal arch arteries (PAAs) and aortic sac arise earlier than those of the posterior PAAs and from a different population of SHF progenitors [23,28]. Thus, while SHF-derived ECs populate the OFT, to our knowledge, it is not known whether they play a critical role in cardiac morphogenesis, or whether *Tbx1*-fated ECs populate the portion of the OFT involved in septation. We investigated this here using mouse genetics approaches.

## 2. Materials and Methods

Mouse lines and genotyping: Mouse studies were performed in accordance with the animal protocol n^o^. 28/2022-PR (approval date: 18 January 2022), reviewed by the local IACUC committee and by the Italian Istituto Superiore di Sanità and approved by the Italian Ministero della Salute, according to Italian law and European guidelines. *Tbx1^Cre/+^* [29], obtained from the European Mouse Mutant Archive (EMMA, Monterotondo, Italy; # EM11399), *Vegfr3^flox/+^* [30], obtained from the EMMA (Monterotondo, Italy; #EM09463), *Vegfr3^+/−^* [12], generated by our laboratory and Rosa*^mTmG^* [31], obtained from Jackson Lab (Bar Harbor, ME, USA; # JAX037456) mice were maintained on a C57BL/6N background. Genotyping of mice was performed as in the original reports.

Immunofluorescence: Embryos were fixed in 4% paraformaldehyde, embedded in paraffin, and sectioned at thickness of 10 µm. The sections were rehydrated, and antigen retrieval was performed in 10 mM sodium citrate pH 6.0. Blocking was carried out using 0.5% milk, 10% fetal bovine serum, and 1% bovine serum albumin H_2_O (blocking solution). Incubation with the primary antibody in blocking solution was carried out overnight at room temperature, and signal detection was performed using secondary antibodies conjugated with different fluorochromes (1:500). We used the following primary antibodies: anti-GFP (ab13970, Abcam Cambridge, UK; 1:1000) and anti-Vegfr3 (14-5988-82, ebioscence/Thermo Fisher Scientific, Waltham, MA, USA; 1:400). The secondary antibodies were: goat anti-Chicken IgY Alexa Fluor™ 488 (A11039 Invitrogen/Thermo Fisher Scientific, Waltham, MA, USA; 1:500) and goat anti-Rat IgG (H + L) Alexa Fluor™ 594 (A11007, Invitrogen; 1:500). Imaging was carried out using using a Leica THUNDER Imaging System (Leica Microsystems, Wetzlar, Germany) equipped with a Leica DFC9000 GTC camera, a Lumencor fluorescence LED light source (Lumencor, Beaverton, OR, USA), and Leica Application Suite X software (version 3.7.4.23463).

RNAscope in situ hybridization: Whole embryos were fixed in 4% paraformaldehyde, dehydrated with increasing concentrations of methanol, and stored at 20 °C. Embryos were hybridized with mRNA probes following the manufacturer’s instructions (RNAscope™, Advanced Cell Diagnostics (ACD), Newark, CA, USA); briefly, whole embryos were rehydrated in decreasing concentrations of methanol and permeabilized for 20 min with Protease III, followed by overnight incubation at 40 °C with the probe solution (see list below). The signal was developed with TSA FITC (Akoya Bioscience, Marlborough, MA, USA, NEL741001KT; 1:500) and TSA-CY3 (Akoya Bioscience NEL745001KT; 1:2000). The embryos were cryoprotected with increasing concentration of sucrose (10%, 20% and 30% sucrose in PBS 1×) at 4 °C, OCT/sucrose 30% (50:50), embedded in OCT, cryosectioned (sagittal and transverse) at a thickness of 10 µm and imaged using the Leica Thunder Imaging System described above. We used the following commercial probes provided by RNAscope™: Probe-Mm-Tbx1-C2 (481911-C2); Probe-Mm-Flt4 (481371). Imaging was carried out as for immunofluorescence experiments.

## 3. Results

### 3.1. Vegfr3 Heterozygosity Is Not a Significant Cause of Cardiac Defects

**Figure 1:**
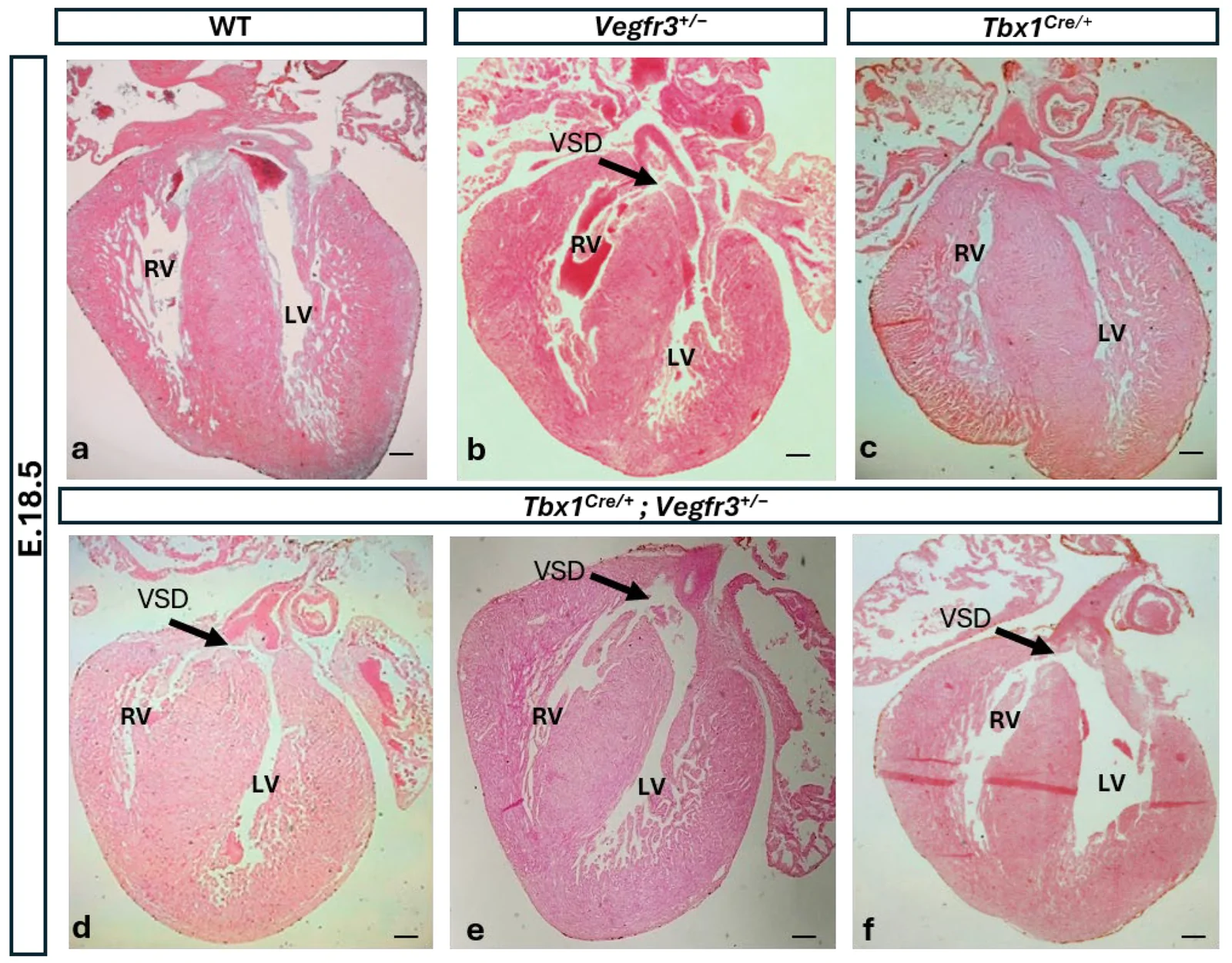
*Tbx1* and *Vegfr3* interact genetically in cardiac development. Coronal sections of isolated hearts, each obtained from a different embryo (E18.5) *WT* (**a**), *Vegfr3^+/−^* (**b**), *Tbx1^Cre/+^* (**c**) and *Tbx1^Cre/+^*; *Vegfr3^+/−^* (**d**–**f**) stained with eosin. Black arrows in panels (**b**,**d**–**f**) indicate the position of a ventricular septal defect (central perimembranous VSD subtype). RV, right ventricle; LV, left ventricle; VSD, ventricular septal defect. Scale bar 100 µm.

To understand whether *Vegfr3* has a role in murine cardiac development, we first evaluated the effect of heterozygous inactivation in the mouse. We analyzed the cardiac phenotype (Table 1, Figure 1) in coronal sections of isolated embryonic hearts at the fetal stage (E)18.5. The results revealed a low penetrance (10%, 1/11) of intraventricular septal defects (VSD) in *Vegfr3^+/−^* embryos compared to WT (0/5) (Figure 1b). The VSD was of the central perimembranous VSD (pmVSD) subtype, i.e., located beneath the aortic valve and with the aorta entirely committed to the left ventricle [32,33]. No other intracardiac or great vessel defects were identified, and the cardiac valves appeared to be normal. We have reported mild cardiac lymphatic vessel hypertrophy in *Vegfr3^+/−^* embryos [12], indicating that in the mouse, loss of one copy of *Vegfr3* affects cardiac lymphatic development but not cardiac morphogenesis.

### 3.2. Tbx1 and Vegfr3 Interact Genetically in Cardiac Development

We have previously shown that in the mouse *Tbx1* and *Vegfr3* interact strongly in brain vascularization and during systemic and cardiac lymphangiogenesis [11,12,13]. To test whether they interact in cardiac development, we intercrossed *Tbx1^Cre/+^* and *Vegfr3^+/−^* mice and analyzed the cardiac phenotype in coronal sections of isolated embryonic hearts at E18.5 (*Tbx1^Cre^* is a null allele) [29]. We found that seven out of eight compound heterozygous embryos analyzed had a perimembranous VSD compared to only one out of eleven *Vegfr3^+/−^* embryos and none out of eight *Tbx1^Cre/+^* embryos analyzed (Table 1 and Figure 1e,f). All the VSDs observed in *Tbx1^Cre/+^*; *Vegfr3^+/−^* embryos were of the central pmVSD subtype. Thus, *Tbx1* mutation greatly enhanced the penetrance of VSD in *Vegfr3^+/−^* mutants, suggesting the existence of a genetic interaction.

### 3.3. Vegfr3 and Tbx1 Are Co-Expressed in the Aortic Sac

**Figure 2:**
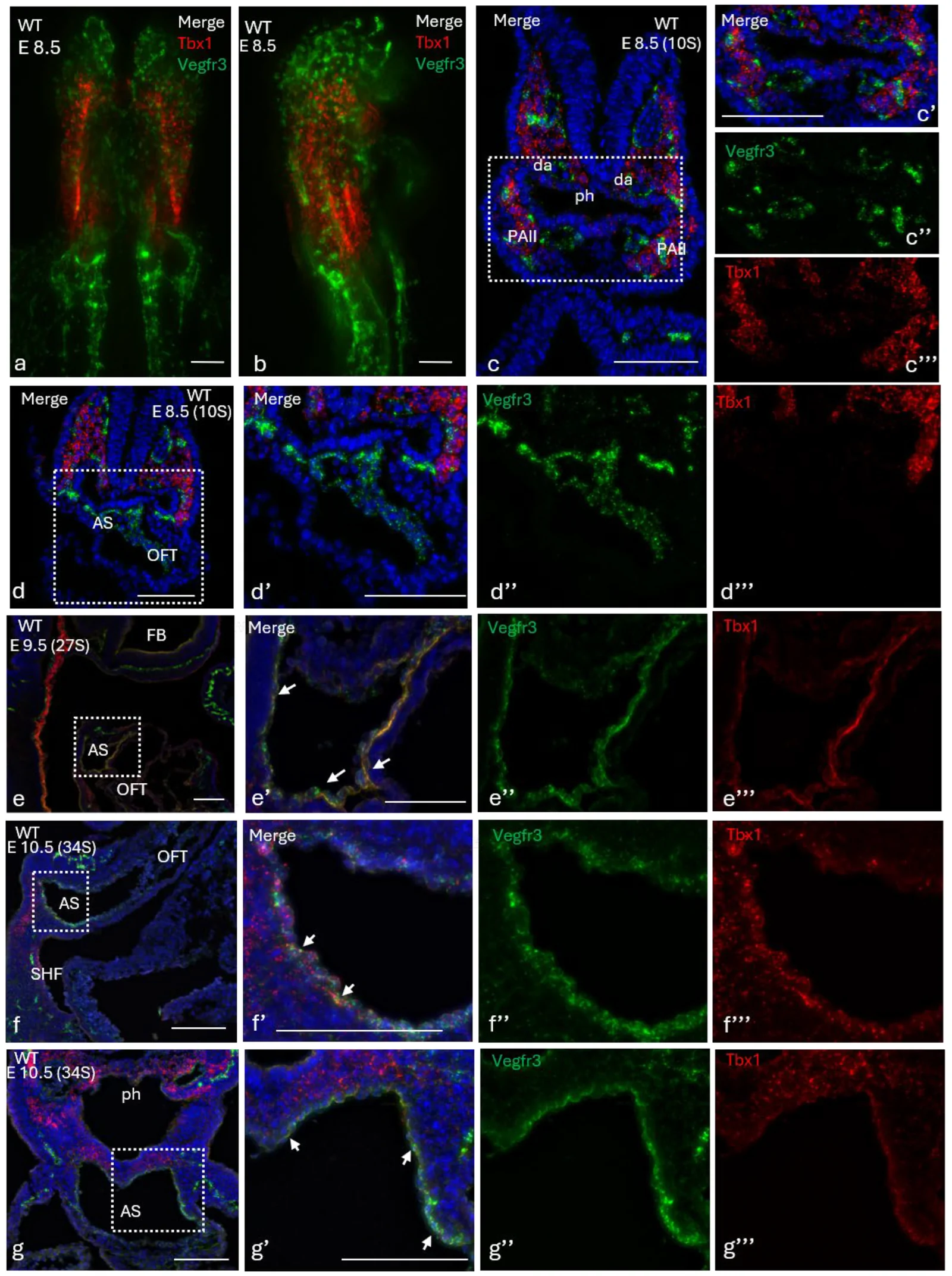
*Tbx1* and *Vegfr3* co-expression in the aortic sac: (**a**,**b**) *Tbx1* (red) and *Vegfr3* (green) expression revealed by RNAscope in wholemount WT embryos at E8.5 with a rostro-caudal orientation (ventral (**a**) and dorsal (**b**) views), and in transverse sections (**c**–**c‴**,**d**–**d‴**) revealed *Vegfr3* but not *Tbx1* expression in the aortic sac. Sagittal sections of WT embryos at E9.5 (27 somites) (**e**–**e‴**), E10.5 (34 somites, (**f**–**f‴**)) and in transverse sections (34 somites, (**g**–**g‴**)) showed that *Tbx1* and *Vegfr3* are co-expressed in the aortic sac. White dashed boxes in (**c**–**g**) indicate the regions shown at higher magnification in the adjacent panels. PAA II, pharyngeal arch artery II; da, dorsal aorta; ph, pharynx; AS, aortic sac; OFT, outflow tract. Scale bar 100 µm.

In order to gain insights into where and when a genetic interaction might occur, we analyzed expressions of *Tbx1* and *Vegfr3* by RNA in situ hybridization using the RNAscope.technology on whole wild-type embryos at E8.5 and E9.5. We then prepared histological sections of the double-stained embryos in order to obtain a detailed view of the distribution of stained cells. Representative embryos are shown in Figure 2. At E8.5, *Vegfr3* was expressed throughout the developing vascular plexus as expected [34], in fully formed blood vessels, including the PAAs (Figure 2a,b) aortic sac (AS) and outflow tract (Figure 2d–d″). At this developmental stage, *Tbx1* was expressed bilaterally in the head mesenchyme (Figure 2c,d), in the core of the pharyngeal arches (PA) I and II (Figure 2c,c‴), and as previously reported in the pharyngeal endoderm and in the second heart field [35]. No double-stained cells (yellow) were detected in any part of the embryo. At E9.5, in the rostral half of the embryo, expressions of *Vegfr3* and *Tbx1* were detected in all of the aforementioned tissues. In addition, *Tbx1* was expressed in endothelial cells lining the AS, but not in the OFT, in embryos with >27 somites (Figure 2e–g). These results indicate that there is no expression overlap between *Tbx1* and *Vegfr3* in the rostral half of the embryo prior to E9.5, but this occurs at the level of the AS between E9.5 and E10.5.

### 3.4. Vegfr3 Is Required in Tbx- Expressing Cells for Normal Cardiac Development

**Figure 3:**
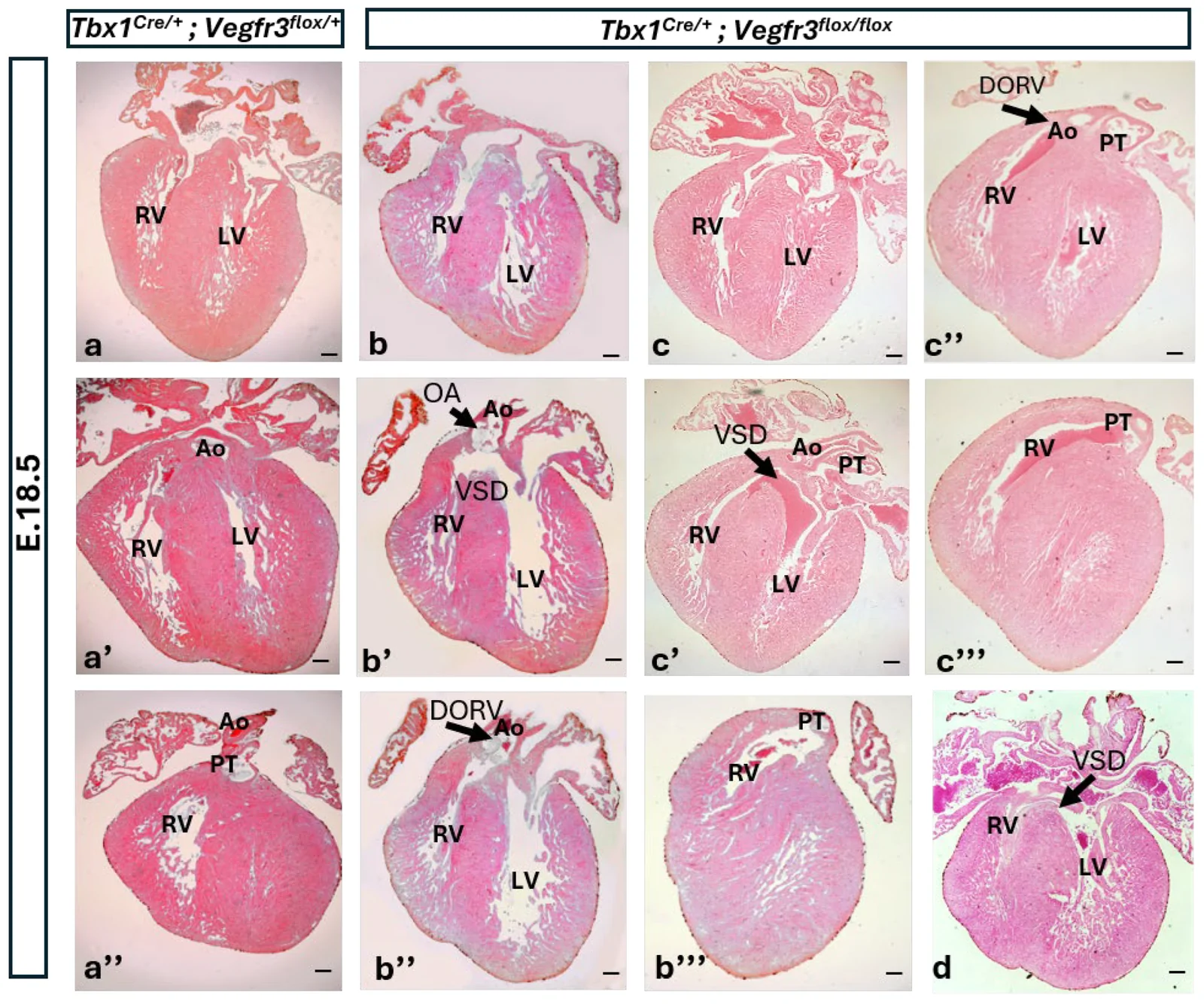
*Tbx1* conditional *Vegfr3* homozygous embryos (*Tbx1^Cre/+^*; *Vegfr3^flox/flox^)* have diverse cardiovascular defects. Coronal sections of isolated hearts of *Tbx1^Cre/+^*; *Vegfr3^flox/+^* (**a**–**a″**) and *Tbx1^Cre/+^*; *Vegfr3^flox/flox^* (**b**–**b‴**,**c**–**c‴**,**d**) embryos (E18.5) stained with eosin. Black arrows in panels (**b′**,**b″**,**c′**,**c″**,**d**) indicate the position of cardiovascular defects. VSD (ventricular septal defect); DORV (double outlet right ventricle); OA (overriding aorta); RV, right ventricle; LV, left ventricle; Ao, aorta; PT, pulmonary trunk. Sections indicated with the same letter are from the same heart. Scale bar 100 µm.

We next tested whether conditional mutation of *Vegfr3* in the *Tbx1* expression domain alone was sufficient to disrupt cardiac development. For this, we intercrossed *Tbx1^Cre/+^*; *Vegfr3^flox/+^* and *Vegfr3^flox/flox^* mice and analyzed the cardiac phenotype in E18.5 embryos (Table 2, Figure 3). Results showed that *Tbx1^Cre^*-driven heterozygous inactivation of *Vegfr3* (thus double heterozygosity in *Tbx1*-expressing cells and their descendants) had no effect on cardiac development (Figure 3a–a″). In contrast, the hearts of *Tbx1^Cre^*-driven conditional *Vegfr3* homozygous embryos (*Tbx1^Cre/+^*; *Vegfr3^flox/flox^*) presented with one or more cardiac defects simultaneously (Figure 3b–d). Cardiac phenotyping of these embryos revealed that VSD was the most prevalent abnormality, being present in six out of seven hearts (85.7%), of which two were classified as isolated central perimembranous VSDs, while the remaining four cases were outlet VSDs with septal malalignment with the great arteries, and they occurred in embryos with double outlet right ventricle (DORV), which was observed in four out of seven hearts (57.1%). Among the DORV cases, two hearts (28.6%) also exhibited an overriding aorta (OA). Finally, we noted that the atrial septum was open in five out of seven hearts (patent foramen ovale, PFO). The FO normally closes after birth with initiation of the pulmonary circulation. Thus, the *Tbx1^Cre/+^*; *Vegfr3^flox/flox^* genotype was associated with variable expressivity of the cardiac phenotype. This result indicates that *Vegfr3* expression in the *Tbx1* expression domain is necessary for normal cardiac development, and that a single active copy of the *Vegfr3* gene in *Tbx1*-expressing cells, or in their progeny, is sufficient for normal development.

**Figure 4:**
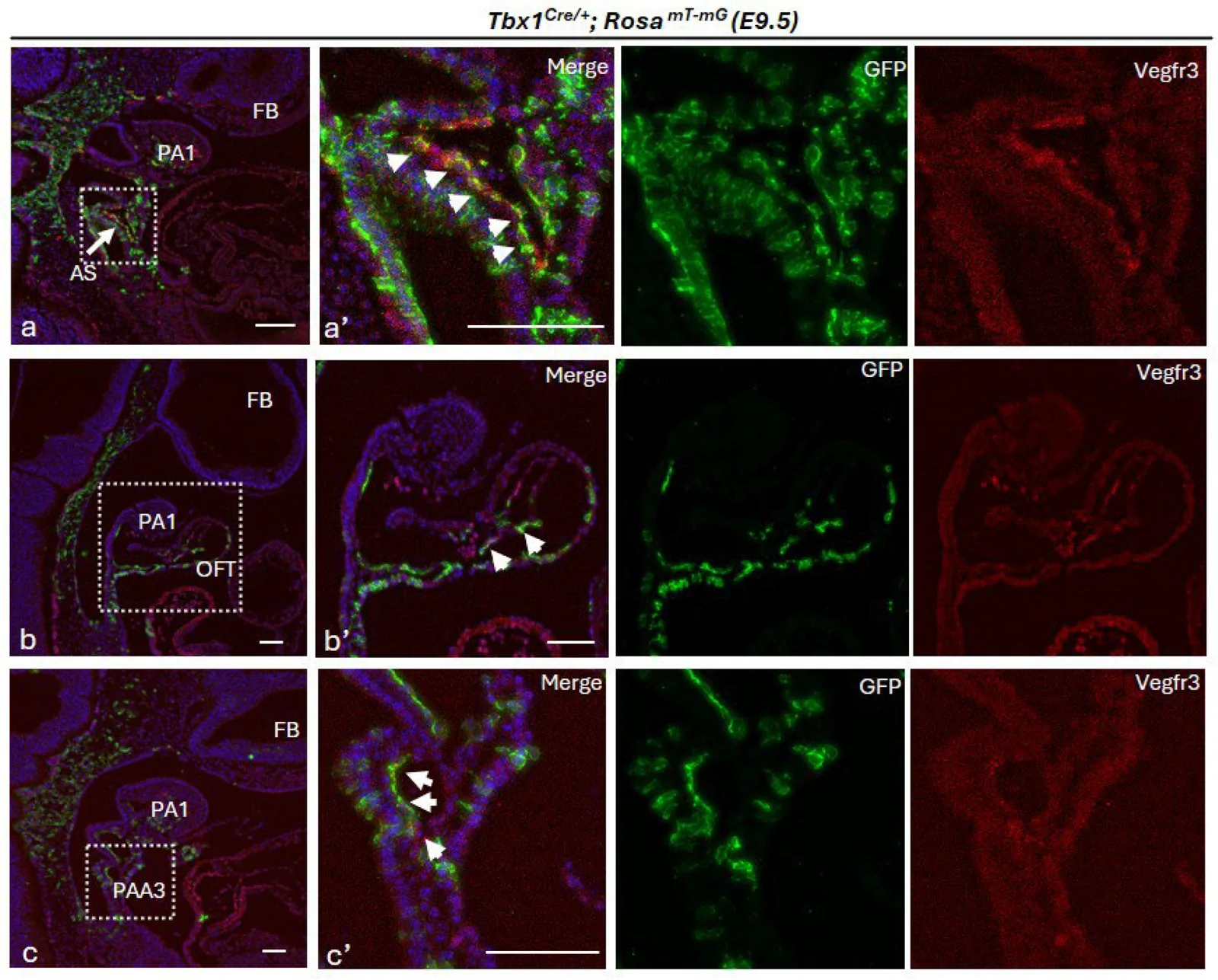
*Tbx1*-activated Cre recombination in *Vegfr3*-expressing endothelial cells: Anti-Vegfr3 (red) and anti-TBX1-GFP (green) immunostaining of sagittal sections of *Tbx1^Cre/+^*; *Rosa^mT-mG^* (E.9.5) reveal *Tbx1*-activated Cre recombination in *Vegfr3*-expressing endothelial cells of the aortic sac (**a**,**a′**), OFT (**b**,**b′**) and third pharyngeal arch artery (PAA3) (**c**,**c′**) (white arrows). White dashed boxes in (**a**–**c**) indicate the regions shown at higher magnification in the adjacent panels. Scale bar 100 µm.

As the endothelium is the only cell type that expresses both *Tbx1* and *Vegfr3*, one would expect that *Tbx1^Cre^*-induced recombination of the *Vegfr3^flox^* allele would recapitulate the phenotype observed in compound heterozygotes (Table 1). However, this was not the case; in fact, *Tbx1^Cre/+^*; *Vegfr3^flox/+^* mutants survived to adulthood, were fertile and had normal hearts (Table 2). In order to better visualize the extent of *Cre* recombination in endothelial cells we performed double immunofluorescence on sagittal sections of E9.5 *Tbx1^Cre/+^*; *Rosa^mTmG^* embryos using anti-GFP and anti-VEGFR3 antibodies. Results showed that in the proximal OFT, the aortic sac and third pharyngeal arch artery, many but not all endothelial cells were double-stained (yellow) (Figure 4a–c′). Thus, there is partial overlap between Cre recombination and VEGFR3 expression in endothelial cells, but evidently, removal of a single copy of the *Vegfr3* gene from this region of overlap is not sufficient to generate cardiac defects.

## 4. Discussion

The results of our work indicate that in the mouse, a single copy of the *Vegfr3* gene is sufficient to support normal heart development in most cases, as only a single case of VSD was observed out of eleven *Vegfr3^+/−^* pre-term embryos. This low frequency might be due to variable expressivity or to stochastic events during pregnancy. A larger study would be necessary to determine whether 10% is the true frequency of VSD in *Vegfr3* heterozygotes. In a recent review by Monaghan and colleagues, the authors proposed that TOF in individuals (human) with *VEGFR3* variants or mutations is not due to *VEGFR3* haploinsufficiency, but rather to the presence of the truncated VEGFR3 protein during fetal development [36]. This deduction was based on published data, showing that: (i) mice carrying *VEGFR3* heterozygous mutations or hypomorphic alleles have normal cardiovascular development [37]; (ii) in humans, truncating mutations are very rare, and thus presumed to be mostly lethal [38,39]; and (iii) in TOF cases with truncating mutations, the mutant protein retained the N-terminal domain but had a shorter C-terminal domain [16]. The authors speculated that truncated VEGFR3 proteins disrupt molecular interactions and cellular functions required for normal cardiogenesis [36]. In our study, the *Vegfr3* mutation is inactivating; therefore, the presence of truncated proteins can be excluded. Thus, the CHDs observed in *Vegfr3^+/−^* and *Tbx1^Cre/+^*; *Vegfr3^flox/flox^* embryos in this study are attributable to gene haploinsufficiency or loss of function. Noteworthy is the observation of *Vegfr3* haploinsufficiency in cardiac lymphatic development [12], suggesting that haploinsufficiency is context-dependent, where different endothelial populations and subtypes have different sensitivities to *Vegfr3* gene dosage.

Our study showed that heterozygous loss of *Tbx1* greatly enhanced the penetrance of VSDs in *Vegfr3^+/−^* mutants from 10% to >85% (Table 1). This might be because TBX1 positively regulates *Vegfr3* in some endothelial cells [11,12,13]; therefore, its mutation might reduce *Vegfr3* expression below a critical threshold required for normal cardiac OFT development, or it might do so indirectly, by disrupting the expression of other *Vegfr3* regulators or by affecting VEGFR3 functions downstream.

Given the strong effect of the *Tbx1* mutation in compound germline *Vegfr3* heterozygotes, we were surprised to find that conditional heterozygous inactivation of *Vegfr3* in the *Tbx1* lineage had no effect on cardiac morphogenesis. We expected that *Tbx1^Cre/+^*; *Vegfr3^flox/+^* and *Tbx1^Cre/+^*; *Vegfr3^+/−^* embryos would have the same, or similar, phenotype, because the *Tbx1^Cre^* allele, which is inactivating [28], is present in both compound genotypes. The difference is likely to be due to incomplete overlap between Cre-induced recombination and *Vegfr3* expression in the critical cell population, which leaves sufficient VEGFR3 to support normal development in *Tbx1^Cre/+^*; *Vegfr3^flox/+^* embryos. Further reduction in VEGFR3 dosage in the homozygous genotype (*Tbx1^Cre/+^*; *Vegfr3^flox/flox^*) is evidently sufficient to trigger disruption of OFT development even in the absence of complete overlap. It is interesting to note that the latter mutants displayed some of the cardinal features of TOF (perimembranous VSD, DORV, OA).

Where might the TBX1-Vegfr3 interaction occur? The RNAscope experiments showed that *Vegfr3* was restricted to ECs, as expected, and that *Tbx1* was expressed in a modest number of ECs in the AS between E8.5 and E9.5. In contrast, when we traced *Tbx1*-expressing cells and their descendants over time (E8.5–E9.5), we observed extensive but incomplete co-localization of the two proteins (GFP of the *Cre*-reporter and VEGFR3) at E9.5 in the AS and in other EC populations of the OFT and pharyngeal arch arteries. Together, these results suggest that *Tbx1* is activated in EC progenitors, consistent with previous in vivo studies [40], destined to contribute to the AS and other structures of the developing cardiovascular system.

In conclusion, this study provides evidence of a regulatory mechanism involving *Tbx1* and *Vegfr3* that is critical for heart morphogenesis. The results of the study point to a two-hit mechanism, whereby reduced dosage of *Vegfr3* in the entire expression domain (not only in the *Tbx1-Vegfr3* overlap domain) has a higher impact on OFT development when combined with *Tbx1* heterozygosity. The interaction between the two genes may be in part direct, in the region of expression overlap, and in part indirect, between the role of *Tbx1* in OFT morphogenesis, weakened by heterozygosity, and the endothelial-dependent role of *Vegfr3*, also weakened by heterozygosity.

At the molecular level, the vascular functions of *TBX1* have been attributed to interactions with molecules in the VEGF-C and Notch signaling pathways [11,12,13]. Previous studies have demonstrated that NOTCH1 signaling in the endocardium is essential for cardiac OFT development [41,42], together with reports identifying dynamic VEGFR3-dependent endothelial populations during cardiovascular morphogenesis [43]. Thus, it is reasonable to think that the regulatory function of TBX1 directly or indirectly affects a NOTCH1-VEGFR3 axis. However, further studies will be required to test directly this hypothesis by dissecting the connections between these molecules.

## Figures and Tables

**Table 1:** Cardiac phenotype in embryos (E18.5) with single and combined genotypes.

Genotype	n	Normal	Cardiac Phenotype
WT	5	5	None
*Tbx1^Cre/+^*	8	8	None
*Tbx1^Cre/+^*; *Vegfr3^+/−^*	8	1	7 VSD (central perimembranous)
*Vegfr3^+/−^*	11	10	1 VSD (central perimembranous)

VSD (ventricular septal defect).

**Table 2:** Cardiac phenotypes in E18.5 embryos with conditional deletion of *Vegfr3* in the *Tbx1* expression domain.

Genotype	n	Normal	Cardiac Phenotype			
*Tbx1^Cre/+^*	8	8	None			
*Tbx1^Cre/+^*; *Vegfr3^flox/+^*	7	7	None			
*Tbx1^Cre/+^*; *Vegfr3^flox/flox^*	7	None	VSD (central pm) 2/7	DORV, outlet VSD 2/7	DORV, OA, outlet VSD 2/7	PFO 5/7

VSD (ventricular septal defect); DORV (double outlet right ventricle); OA (overriding aorta); PFO (patent foramen ovale).

## Data Availability

All data are contained within the manuscript.

## Abbreviations

OFT	outflow tract
Vegfr3	vascular endothelial growth factor receptor 3
DGS	DiGeorge syndrome
CPM	cardio-pharyngeal mesoderm
AS	aortic sac
SHF	second heart field
PAAs	pharyngeal arch arteries
RV	right ventricle
LV	left ventricle
R26R	Rosa26 reporter
VSD	ventricular septal defect
WT	wild type
PA	pharyngeal arches
DORV	double outlet right ventricle
OA	overriding aorta
PFO	patent foramen ovale
PT	pulmonary trunk
CHD	congenital heart disease
TOF	Tetralogy of Fallot
